# An estrogen-induced endometrial hyperplasia mouse model recapitulating human disease progression and genetic aberrations

**DOI:** 10.1002/cam4.445

**Published:** 2015-03-23

**Authors:** Chieh-Hsiang Yang, Aliyah Almomen, Yin Shen Wee, Elke A Jarboe, C Matthew Peterson, Margit M Janát-Amsbury

**Affiliations:** 1 Division of Gynecologic Oncology, Department of Obstetrics and Gynecology, University of UtahSalt Lake City, Utah, 84132; 2 Department of Bioengineering, University of UtahSalt Lake City, Utah, 84112; 3 Department of Pharmaceutics and Pharmaceutical Chemistry, University of UtahSalt Lake City, Utah, 84112; 4 Division of Microbiology and Immunology, Department of Pathology, University of Utah School of MedicineSalt Lake City, Utah, 84124; 5 Department of Pathology, University of UtahSalt Lake City, Utah, 84112

**Keywords:** Endometrial cancer, endometrial hyperplasia, estrogen, mouse model

## Abstract

Endometrial hyperplasia (EH) is a condition originating from uterine endometrial glands undergoing disordered proliferation including the risk to progress to endometrial adenocarcinoma. In recent years, a steady increase in EH cases among younger women of reproductive age accentuates the demand of therapeutic alternatives, which emphasizes that an improved disease model for therapeutic agents evaluation is concurrently desired. Here, a new hormone-induced EH mouse model was developed using a subcutaneous estradiol (E2)-sustained releasing pellet, which elevates the serum E2 level in mice, closely mimicking the effect known as estrogen dominance with underlying, pathological E2 levels in patients. The onset and progression of EH generated within this model recapitulate a clinically relevant, pathological transformation, beginning with disordered proliferation developing to simple EH, advancing to atypical EH, and then progressing to precancerous stages, all following a chronologic manner. Although a general increase in nuclear progesterone receptor (PR) expression occurred after E2 expression, a total loss in PR was noted in some endometrial glands as disease advanced to simple EH. Furthermore, estrogen receptor (ER) expression in the nucleus of endometrial cells was reduced in disordered proliferation and increased when EH progressed to atypical EH and precancerous stages. This EH model also resembles other pathological patterns found in human disease such as leukocytic infiltration, genetic aberrations in *β*-catenin, and joint phosphatase and tensin homolog/paired box gene 2 (PTEN/PAX2) silencing. In summary, this new and comprehensively characterized EH model is cost-effective, easily reproducible, and may serve as a tool for preclinical testing of therapeutic agents and facilitate further investigation of EH.

## Introduction

Endometrial cancer (EC) still ranks as the most common gynecological malignancy and, despite the low case-fatality ratio, causes more than 8000 deaths per year in the United States alone 1. Endometrioid adenocarcinoma is the most frequently diagnosed EC subtype accounting for 80–90% of all cases. This subtype is often preceded by a precursor lesion, atypical endometrial hyperplasia (EH) also known as endometrial intraepithelial neoplasia (EIN), and is associated with a continuous estrogen stimulation over time commonly referred to as estrogen dominance 2–4. When diagnosed in a woman after her childbearing, reproductive years, hysterectomy typically serves as the standard treatment. But in younger patients this is often not an acceptable approach, demanding an alternative, nonsurgical management 5,6. Since a steady increase in EH incidence among younger women has been observed, therapeutic alternatives are mandated 7. The development of an effective, alternative method to treat EH requires an improved understanding of mechanisms and pathways involved in the development and progression of EH to EC.

Advancement of new therapeutic agents is often hampered by the lack of suitable preclinical in vivo EH models with which researchers can investigate the course of the disease, assess genetic variations, as well as evaluate therapeutic response. In an attempt to closely mimic human EH, genetically engineered mouse models (GEMMs) and carcinogen-induced mouse models have been established 8–14. Although GEMMs facilitate the study of the mechanisms in disease progression, GEMMs is defaulted by either a mutation or deletion of a single-potent oncogene, which may consequently fail to recapitulate the genetic variations present in the actual human condition 15,16. Besides, GEMMs can be economically challenging as well as time-consuming since they can take over 26 weeks to develop the disease 13,14.

In contrary to GEMMs, carcinogen-induced mouse models utilizing either chemical compounds or hormones are less time-consuming and less cost-intensive 11,12. Hormone-induced models may actually be more representative of the condition observed in human patients regarding the mechanism and disease progression of EH. It has been proven that feeding mice with high daily doses of E2 can induce EH in mouse uteri within 3 days 9. However, oral gavage, the method of choice for EH induction, is often accompanied by complications such as esophageal reflux, aspiration pneumonia, esophageal, and gastric rupture, besides being stressful to the animals 17. Such procedural complexity may limit the wider use as well as disease reproducibility for broader application in therapeutic development and screening. Short-term induction of the disease in this model may not allow comprehensive characterization of the processes involved in EH progression including crucial stages of onset of tumorigenesis. Further, clinically relevant hormone receptor status and genetic aberrations associated with EH developed in mice remain unaddressed.

In this study, we report the establishment of an E2-induced EH mouse model using a subcutaneous E2-sustained release implant, which by mechanisms of controlled drug release lead to an elevated serum E2 levels in mice closely resembling the etiology of EH in human patients. This model is the first comprehensively characterized EH model, which recapitulates clinically relevant events including the chronologic development of EH starting from normal, healthy endometrium transforming to EH, and then progressing to precancerous stages. Upon the onset of different stages of EH, crucial histological changes including hormone receptors alteration, leukocytic infiltration, and genetic aberrations were observed and evaluated.

## Materials and Methods

### Preparation and characterization of a controlled-release E2 delivery system

#### Preparation of an E2-sustained release delivery system in form of pellets

E2 pellets were prepared as previously described 18. Briefly, 3.9 g of beeswax (Sigma-Aldrich, St. Louis, MO) was placed into a 20-mL glass sample vial (Thermo Fisher Scientific Inc., Waltham, MA) and melted on the hot plate at 65°C. Then 100-mg *β*-Estradiol (Sigma-Aldrich) was added to the melted wax and mixed using a sterile 7-mm magnetic stir bar until the mixture turned clear. A sheet of aluminum foil was placed on top of the dry ice and the wax/*β*–Estradiol mixture was dropped onto the foil using a preheated glass Pasteur pipet to create a pellet. Each pellet was made from three drops of the wax/*β*–Estradiol mixture, resulting in ∼30 mg per pellet. Pellets were stored at 4°C for further study.

#### In vitro E2 release kinetics from pellets

A single E2 pellet was placed in a 20-mL glass sample vial and 1 mL of pre-warmed (37°C) 1xPBS (phosphate-buffered saline) was added. The sample vial containing the E2 pellet was incubated at 50 rpm, 37°C for 0.5, 1, 2, 4, 6, 8, 12, 24, 48, 72, 96, 120, 144, 168, 336, and 672 h. At each time point, 1xPBS was collected and replaced with 1 mL of fresh prewarmed (37°C) 1xPBS. The sample was then mixed with 2-mL ethyl ether (Mallinckrodt Chemicals, Cary, NC), vigorously vortexed for 15 sec and incubated at −20°C until complete freezing of the aqueous phase. The organic layer (upper layer of ethyl ether) was collected in a 15-mL conical tube and placed in a fume hood until complete evaporation of the organic phase. The residue was then suspended in 1-mL DMSO (Sigma-Aldrich), and the amount of E2 was measured at 284 nm on a Varian Cary 400 Bio UV-visible spectrophotometer (BioTech, MD, USA). Quantitative determination of E2 was conducted using a linear calibration curve between the range of 0.78–25 *μ*g/mL with a 0.99 correlation coefficient.

### Animals and EH induction

#### Subcutaneous implantation of E2 pellets

About 6–8 week-old female Balb/c mice were purchased from Jackson Laboratories (Bar Harbor, ME). Animals were housed in the Animal Facility of the Comparative Medicine Center at the University of Utah under standard conditions and all procedures were conducted following approved Institutional Animal Care and Use Committee (IACUC) protocols.

A total of 30 mice were used for E2 pellet implantation and randomly divided into six groups (*n* = 5). Animals total body weights were recorded before the surgical procedure and each mouse was anesthetized with a mixture of 1 mL of ketamine (Vedco Inc., Saint Joseph, MO), 0.1 mL of xylazine (LLOYD, Shenandoah, IA), and 8.9 mL of normal saline. Mice received intraperitoneal injections of ketamin–xylazine mixture combined with subcutaneous buprenorphine (Reckitt Benckiser Pharmaceuticals, Richmond, VA). Doses were adjusted accordingly to each animal body weight. A small incision, ∼2–3 mm in length, was made between the shoulder blades to form a subcutaneous pocket. One E2 pellet was inserted into the pockets of each animal. Incisions were closed using 4-0 polyglycolic acid suture with a taper needle (Ethicon, Somerville, NJ) and then disinfected with alcohol/iodine.

#### Tissue harvest and storage

Mice were sacrificed at 2, 4, 6, 8, and 10 weeks postimplantation of E2 pellets to assess local and systemic hormone effects. Mouse uteri were fixed in 4% paraformaldehyde for 24–48 h and then paraffin embedded. Blood samples were centrifuged at 200 g, 4°C for 10 min, and serum was collected and stored at −80°C prior to estradiol enzyme immune assays.

#### Serum E2 analyses

Serum was pooled from five mice representing each time point (2, 4, 6, 8, and 10 weeks postimplantation of E2 pellets) and triplicate samples were assayed using an estradiol enzyme immune assay kit (Cayman, Ann Arbor, MI, USA) following manufacturer instructions.

### Histology evaluation

#### Hematoxylin and eosin staining

Tissue sections were deparaffinized in xylene, rehydrated in descending ethanol series, and then stained in Mayer’s hematoxylin (Sigma-Aldrich) for 3 min. Following rinsing in 0.1% HCl solution and tap water, sections were stained in eosin stain for 1–2 min and then rinsed in tap water. Sections were dehydrated in ascending ethanol series, cleaned with xylene, and mounted. Gland-to-stroma ratio was quantified by randomly selection of 10 fields from five mice using Image J 1.44o software (N.I.H., Bethesda, MD, USA).

#### Immunohistochemical staining

To minimize the variability, which may potentially affect the result of staining, all slides were stained at the same time by the same researcher. Staining was performed on 5-*μ*m tissue sections obtained using a standard microtome. Sections were deparaffinizied and rehydrated following conventional procedures. Antigen retrieval was then conducted in 10-mM sodium citrate buffer (pH 6.0). Sections were incubated in peroxidase blocking reagent provided in Dako EnVision kit (Dako, CA, USA) following blocking buffer for 1 hr. After blocking buffer was removed, primary antibody was applied on the sections. Sections were washed in PBS-T and incubated with secondary antibody for 30 min at room temperature. Stains were visualized by 3,3-diaminobenzidine (Dako), with Mayer’s hematoxylin (Sigma) as a counter stain, and mounted. Slides were read and evaluated by a board certified clinical pathologist. The detailed antibody information and staining conditions are listed in Table S1.

### Statistical analysis

Statistical analysis and plotting graphs were performed using GraphPad Prism software (GraphPad Software Inc. San Diego, CA, USA). All experiments are conducted in triplicate unless indicated otherwise. Results are expressed as mean ± SEM, and P < 0.05 was considered significant.

## Results

### E2 pellets exhibited sustained release kinetics in vitro

**Figure 1:**
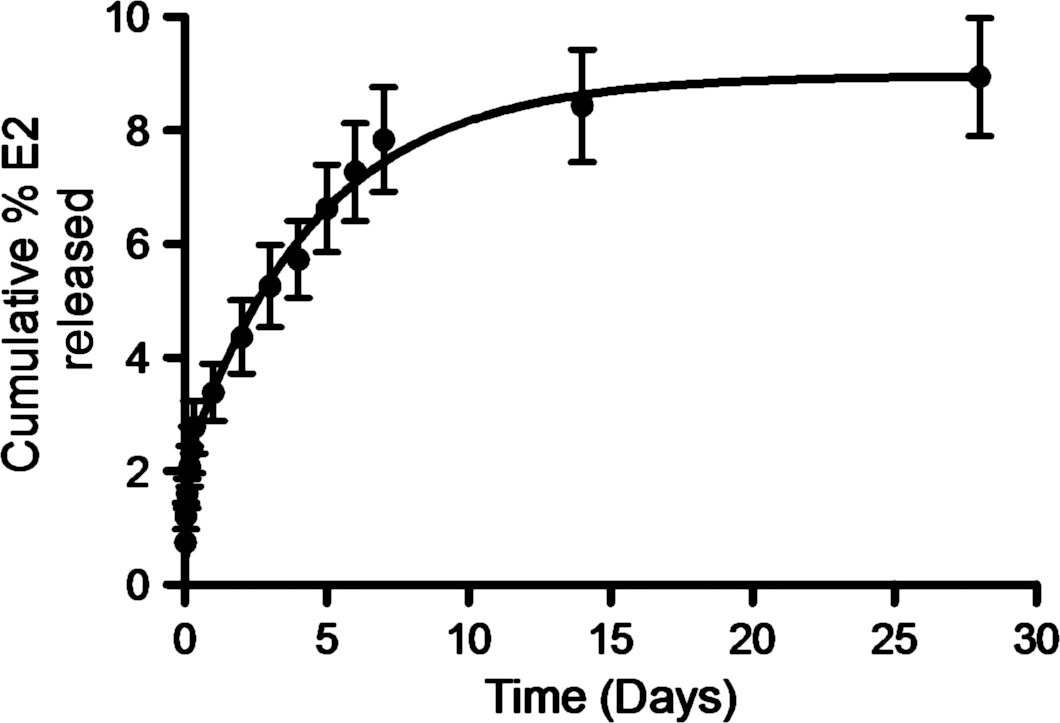
In vitro release kinetics of E2 pellets. The release profile of E2 from wax pellets showed a linear increase in the drug release within the first 7 days of pellet exposed to release media followed by a sustained release rate. (*N* = 5, mean ± SEM).

In an attempt to characterize the release function of wax-based E2 pellets and better mimic the biological condition of mice, E2 pellets were incubated in prewarmed (37°C) 1xPBS at 50 rpm for 28 days. At different time points, sample was collected and replaced with fresh prewarmed 1xPBS. The release profile of E2 from wax pellets showed a linear increase in the drug release within the first 7 days. After 7 days, release kinetics reached a plateau phase, exhibiting a sustained release rate to the end of the tested time point. A total of ∼10% E2 was released within 28 days (Fig.1).

### E2 levels in mouse serum were elevated to pathological levels after implantation of E2 pellets

**Figure 2:**
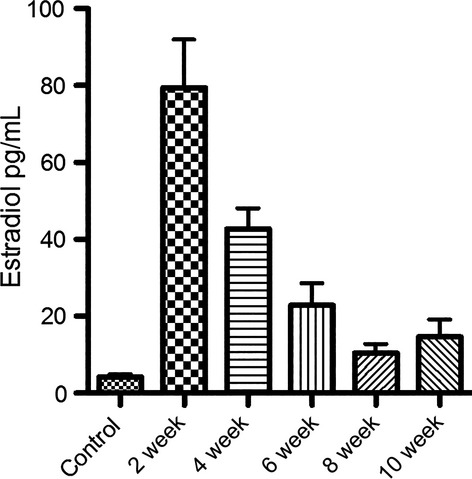
E2 levels in mouse serum at 2, 4, 6, 8, and 10 weeks post-implantation of E2 pellets. Peaked E2 levels were reached within 2 weeks after pellet implantation and serum E2 concentrations at all time points were at least twofold higher than controls. (*N* = 5, mean ± SEM).

To evaluate the affect of E2 pellets on elevating mouse serum E2 levels, mouse serum samples were collected at the time of study endpoints. E2 serum levels after the implantation of the pellets are shown in Figure2. Peaked E2 levels (79.42 ± 12.51 pg/mL) were reached within 2 weeks after pellet implantation. E2 concentrations at each time point were at least twofold higher when compared to controls (E2 nontreated mice).

### Mouse uteri revealed the development of atypical hyperplasia following E2 exposure

**Figure 3:**
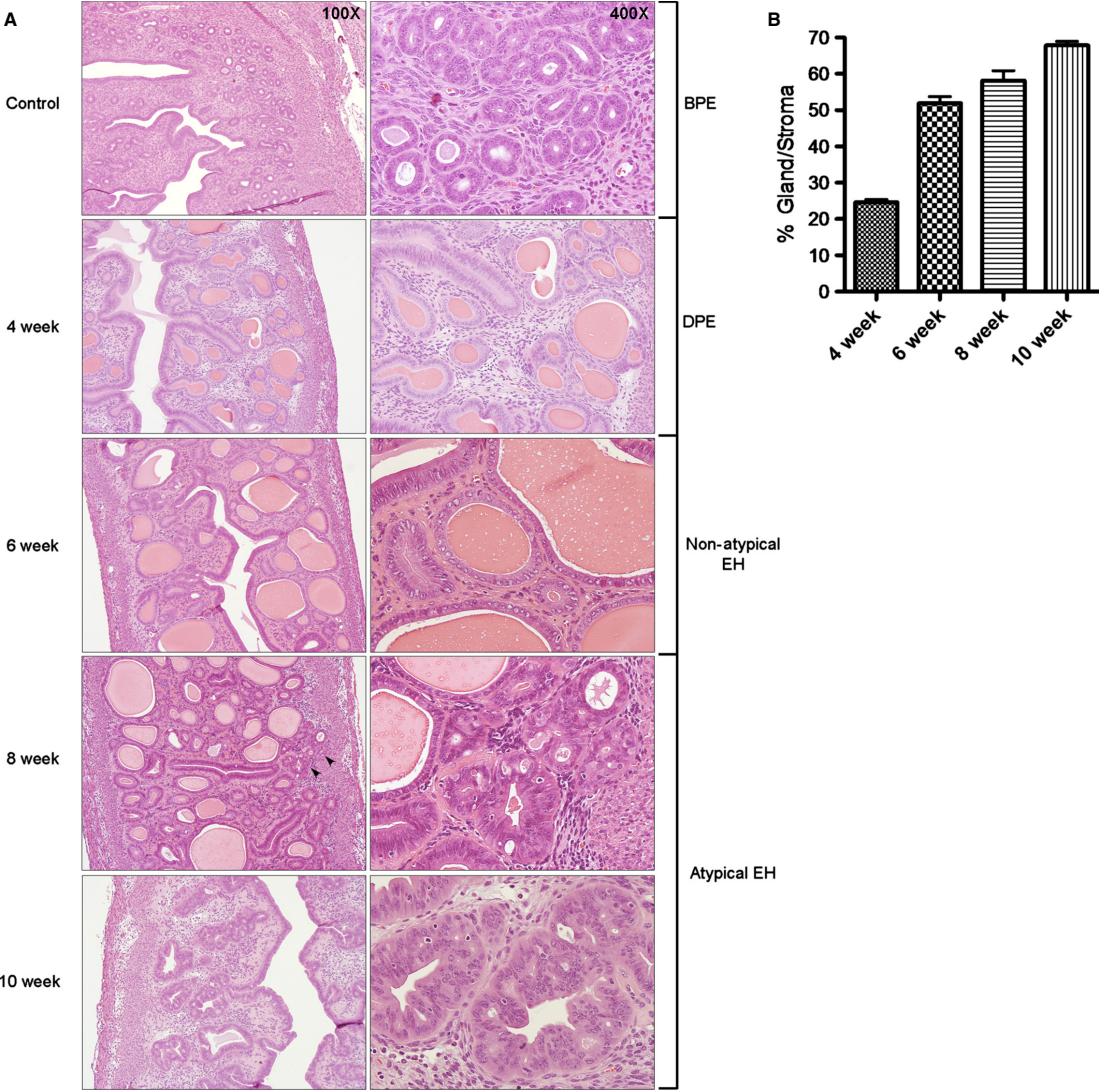
Progression to atypical hyperplasia in mouse uteri after expose to E2. (A) Benign proliferative endometrium (BPE) featuring small, tubular glands, which were regularly spaced in abundant stroma at control group (100×). The cells lining the glands were predominantly basally oriented, with brisk mitotic activity (400×). Disordered proliferative endometrium (DPE) was presented at 4 weeks post-implantation of E2 pellets and is characterized by scattered, cystic glands in an otherwise normal-looking endometrium (100× and 400×). At 6 weeks, nonatypical hyperplasia was presented and featured irregular gland density. Glands are variable in shape; many are cystic (100×). Cytologically, the glandular cells were similar to those of BPE (400×). After 8 weeks E2 exposure, localized atypical EH was presented (black arrow). The focus exhibited closely packed glands, some of which were irregularly shaped (100×); nuclear atypia was identified, characterized by nuclear stratification with loss of polarity, irregularly shaped nuclei, and prominent nucleoli (400×). Atypical hyperplasia was diffusely presented at 10 weeks group (100× and 400×). (B) Gland-to-stroma ratio of mouse uteri were quantitatively analyzed and compared between groups. Gland-to-stroma ratio exceeded 50% after 6 weeks of E2 exposure. (*N* = 5, mean ± SEM).

To confirm capability of mouse uteri to develop EH in a clinically relevant pattern after the exposure to E2, mice were sacrificed and uteri were examined using hematoxylin and eosin (H&E) staining at the aforementioned time points. Histological evaluation of mouse uteri after 2 weeks of E2 exposure revealed a proliferative endometrium with a normal gland-to-stroma ratio. Minimal cystic dilation was noted in rare glands, in the absence of any gland crowding or other cytologic alterations (data not shown). After 4 weeks postimplantation, features of disordered proliferative endometrium were recognized: scattered cystic glands were present; gland crowding was not noted, and the cells lining the cystic glands were cytologically identical to those in the normal background proliferative endometrium (Fig.3A). The endometrium at 6 weeks of E2 exposure was remarkable for a variable density of proliferative endometrial glands, present in a “regularly irregular” distribution. Glands exhibited variability in shape as well, suggesting that endometrial cells underwent polyclonal proliferation. Cytologically, there was no difference between glands in crowded and uncrowded areas. These findings were characteristic of nonatypical hyperplasia and the gland-to-stroma ratio of mouse endometrium exceeded 50% (Fig.3B). Atypical hyperplasia was apparent at 8 weeks and 10 weeks of postimplantation of E2 pellets. Discrete areas of marked gland crowding were present. These crowded glands were irregular in shape and were lined by cells exhibiting nuclear atypia, characterized by loss of nuclear polarity, irregular nuclear shape, and prominent nucleoli. These changes were present focally in the endometrium at 8 weeks, and more diffusely at 10 weeks. The success rates for the development of different stages of EH based on time of exposure to E2 are described in Table S2.

### Expression of hormone receptors were altered as the EH advanced to atypical hyperplasia

**Figure 4:**
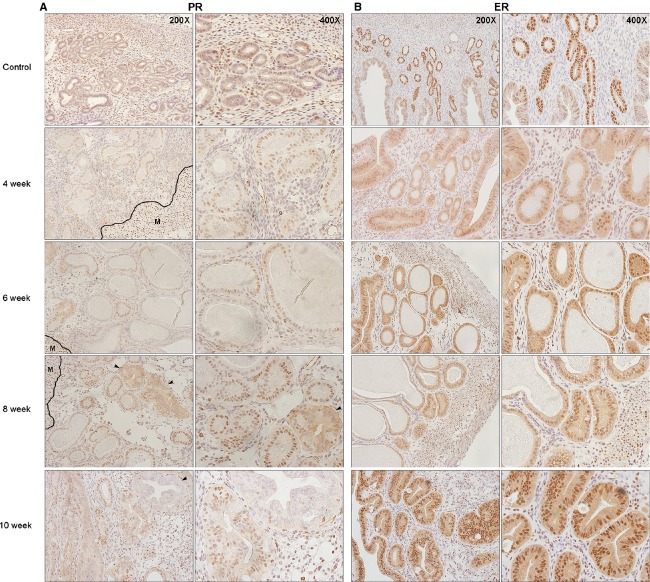
Alterations of hormone receptors expression in mouse uteri after expose to E2. (A) PR expression increased after E2 exposure. At 6 weeks, loss of PR expression in the endometrial cells was observed. Diffused cytoplasmic expression was observed in atypical gland at 8 weeks after E2 exposure (black arrow). However, a complete loss of PR expression in some atypical hyperplastic glands occurred in 10 weeks group (black arrow). Myometrium served as an internal staining control and showed strong positive nuclear staining. M: myometrium. (B) ER expression in endometrium was significantly reduced after 4 weeks of E2 exposure and increased at 6 weeks. PR, progesterone receptor; ER, estrogen receptor.

To examine whether hormone receptors expression in our model recapitulate status in clinic, we evaluated expression levels of both estrogen receptor (ER) and progesterone receptor (PR). The intensity of PR expression was stronger in hyperplastic endometrium compared to normal proliferative endometrium, and was found to be the strongest in the atypical type (Fig.4A). Loss of PR expression in the endometrial cells was first observed in 6 weeks after E2 exposure. Further, a diffused cytoplasmic expression of PR was observed in atypical EH. As atypical hyperplasia advanced to borderline cancer, an obvious loss of PR expression in the glandular epithelium occurred. Although, an initial reduction in ER expression in the glandular epithelium was found after 4 weeks of E2 exposure relative to normal proliferative endometrium, an increased in ER expression occurred in hyperplastic endometrium and was the strongest in the atypical cells (Fig.4B).

### Leukocytic infiltration occurred as the glandular epithelium transformed into atypia

**Figure 5:**
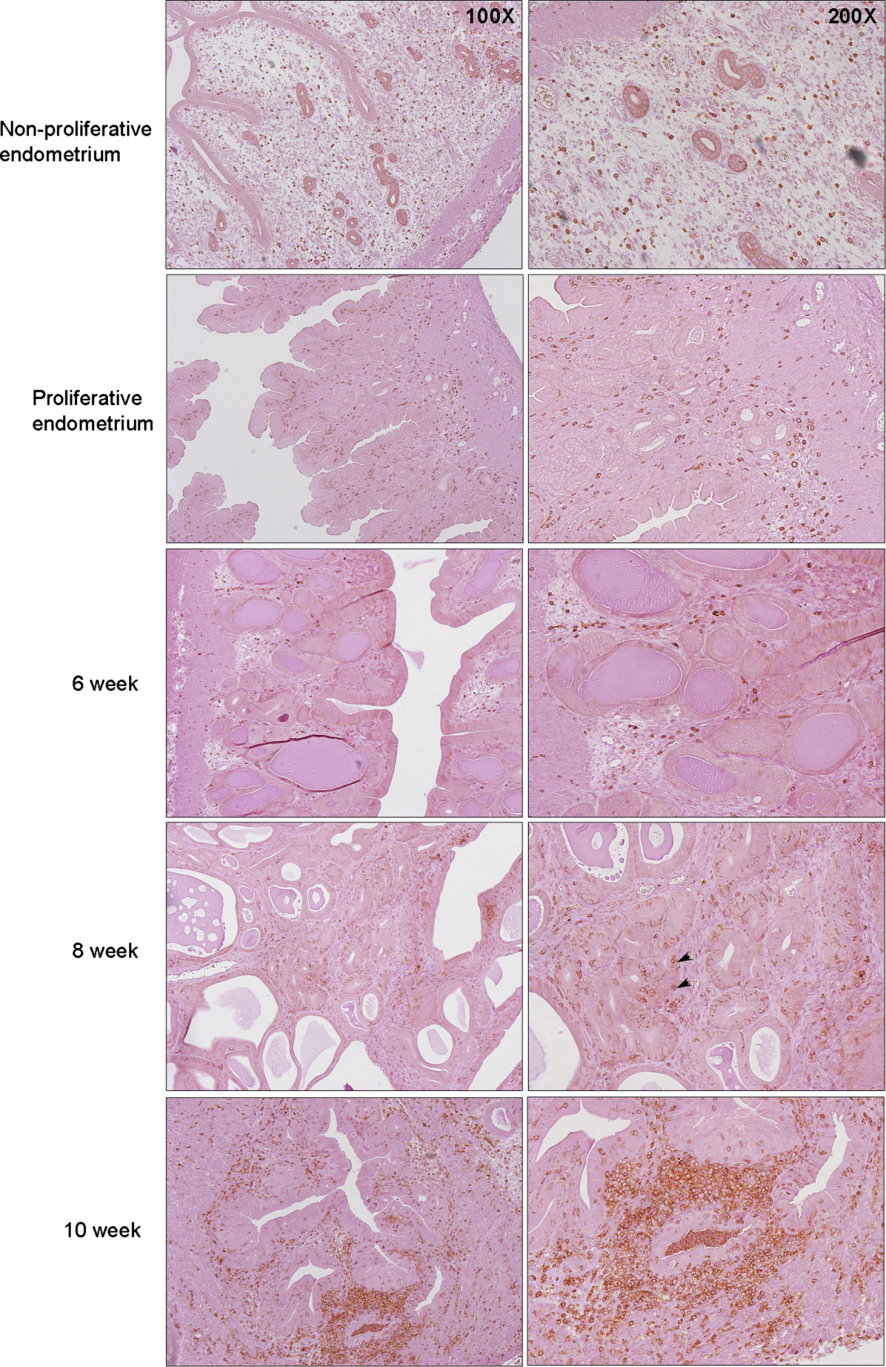
Leukocytic infiltration occurred as the glandular epithelium transformed to atypia. CD45+ cells were homogenously scattered throughout the endometrial stroma in normal nonproliferative endometrium. In the proliferative phase and nonatypical hyperplasia (6 weeks group), CD45+ cells increased in number, but still maintained their homogenous distribution. Penetrations of CD45+ cells into localized atypical glands occurred at 8 weeks of E2 exposure (black arrow). In atypical EH at 10 weeks, the number of CD45+ cells increased significantly, and the atypical glands were surrounded and penetrated by these cells.

Estrogen is known to be involved in the production of proinflammatory components, which might participate in the neoplastic transformation, and carcinogenesis of the endometrium. Further, it was reported that CD45+ cells could be involved in the proliferative action induced by E2. Here, CD45+ cells were homogenously scattered throughout the endometrial stroma in the normal nonproliferative endometrium. In the proliferative phase, CD45+ cells start aggregating around the glandular epithelium. The number of stromal CD45+ cells increased in nonatypical hyperplasia, but still maintain their homogenous distribution. The penetration of CD45+ cells into localized atypical glands starts to occur within 8 weeks of E2 exposure. However, a marked increase in CD45+ cells surrounding the glands and infiltration to the glandular epithelium occurred in atypical EH after 10 weeks of E2 exposure (Fig.5).

### Clinically relevant genetic aberrations were induced in the mouse model

**Figure 6:**
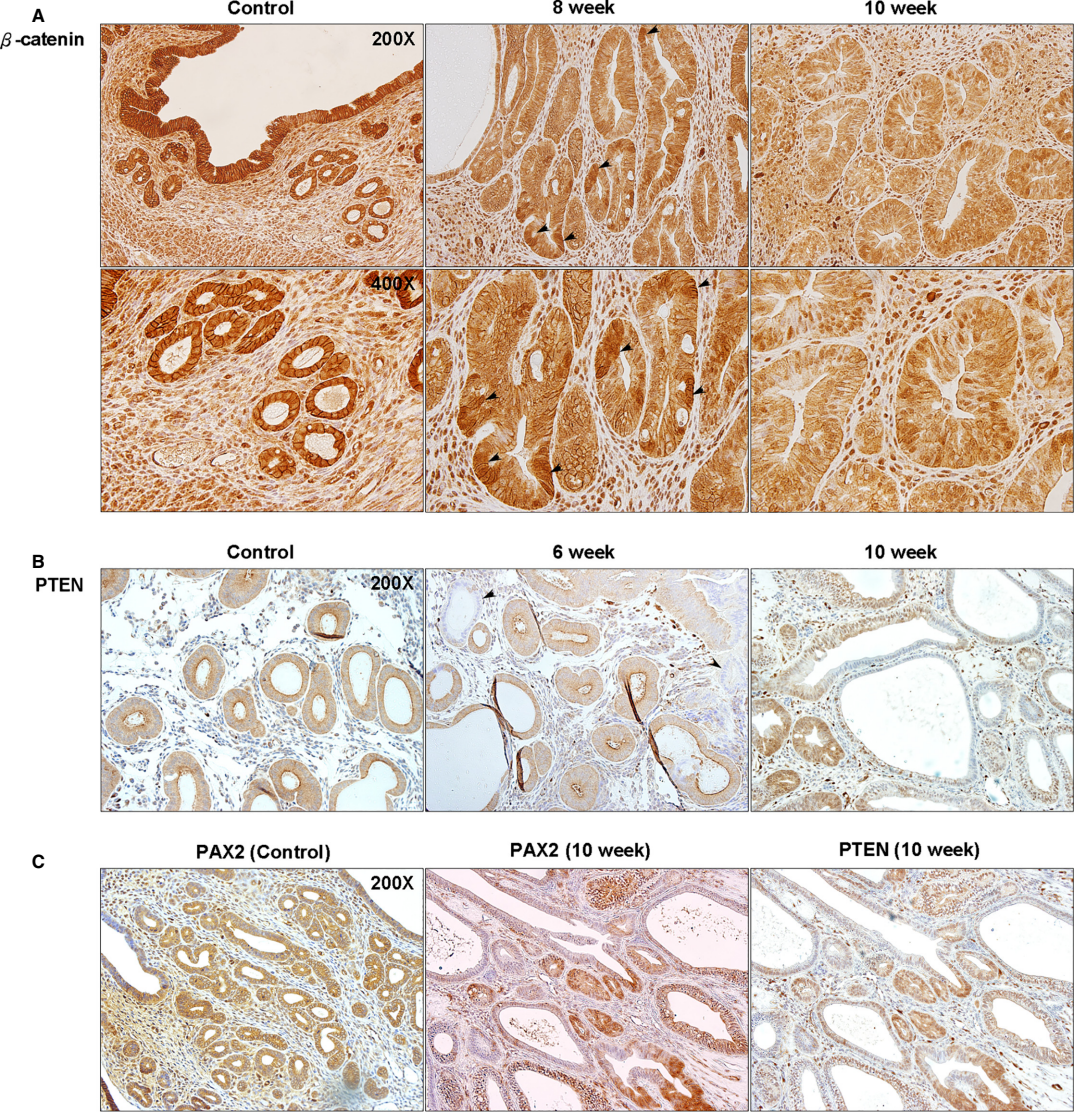
Aberrations of *β*-catenin, PTEN, and PAX2 in atypical EH of mouse uteri. (A) In normal proliferative endometrium, *β*-catenin expression was in the cytoplasm with a homogenous pattern. At 8 weeks of E2 exposure, the accumulation of *β*-catenin started to present in the diffused atypical glands. At 10 weeks, the pattern of *β*-catenin expression became heterogeneous and started to present in the nucleus of endometrial cells. (B) Loss of PTEN was observed as early as 6 weeks after E2 exposure, even in glands with normal appearance compared to control. At 10 weeks, more glands with PTEN silencing presented and the expression pattern started to loss its homogeneity. (C) PAX2 silencing was also observed and presented an expression that is a similar to PTEN. PTEN, phosphatase and tensin homolog; PAX2, paired box gene 2.

To investigate the capability of our model in replicating genetic aberrations mostly seen in human EH, expression of *β*-catenin, phosphatase, and tensin homolog (PTEN), and paired box gene 2 (PAX2) were evaluated using immunohistochemical staining. In a normal endometrium, regardless of the cycle phase, *β*-catenin expression presents homogeneous patterns only in the cytoplasm of the glandular epithelium. Our results showed an accumulation and a heterogeneous expression of *β*-catenin in the diffused atypical glands, which was limited to the cytoplasm. However, a nuclear expression of *β*-catenin was seen in the 10 weeks group (Fig.6A). Loss of PTEN expression in the glandular epithelium started within 6 weeks of continuous exposure to E2 and, a complete loss of PTEN in some of the glands with an atypical appearance became more intense in 10 weeks group (Fig.6B). A concurrent PAX2 silencing was also observed and presented an expression that is similar in pattern to PTEN (Fig.6C).

## Discussion

EH remains a serious health problem in the developed countries, requiring immediate attention to improve the management of the disease, especially for women who are still in their childbearing age. Development of new treatment approaches for EH still lacks a reliable and easily reproducible in vivo animal model. Here, we report the establishment of a new EH mouse model using an E2 sustained releasing subcutaneous implant. This model recapitulates the actual onset and disease course of EH originating from healthy normal endometrium, to disordered proliferation, advancing to nonatypical EH, further progressing to atypical EH, and finally leading to the development of borderline EC. This animal model goes in parallel with the clinical scenario including the alterations of hormone receptors expression and leukocytic infiltration. This model also succeeded in generating various clinically relevant genetic aberrations including *β*-catenin, PTEN, and PAX2. Therefore, our EH model indicates that it is capable to represent different patient populations clinically presenting with EH. Based upon its genetic variety, this model may also have implications to study and gain a better understanding of the etiology underlying EH and its transition to EC.

Although the use of wax-based pellets was previously described on supporting the growth of ER+ breast tumor xenograft and enhancing the effect of EC potent oncogene, this is the first report utilizing this technique for generating endometrial tissue abnormalities 19,20. In order to closely imitate EH in premenopausal women, oophorectomy was not performed on mice in this study. This strategy allows to better reflect pathophysiological conditions seen in human EH caused by the exposure of uterine tissues to continuously elevated E2 levels over extended periods of time. A steady plateau level of E2 was reached in the mouse serum after 6–8 weeks post-pellet implantation. In all cases, the concentration of E2 in mouse serum was at least twofold higher than physiological levels in control mice resembling the fold increase in plasma E2 in EH patient 21,22. A drop of the E2 concentration in the serum was observed within few weeks after pellet implantation, which may be attributed to the induction of cytochrome p450 by E2. The induction of cytochrome p450 enhances the metabolism and elimination of E2 and reduces its serum levels 23. In addition, E2 can also induce the production of sex hormone-binding globulin thus reducing the amount of free E2 in the blood 24,25.

To examine whether EH can be induced in other stains of mice, we implanted E2 pellets into both immunocompetent and immunocompromised mice in addition to Balb/c mice. All immunocompromised mice, including Nu/Nu, NOD/SCID, and NOD-scid IL2R*γ*^null^, developed atypical EH after 10 weeks post-implantation. However, animals developed cystitis after 8 weeks of E2 exposure, which itself became a limiting complication, affecting sustainability of EH modeling in these particular mouse strains. C57BL/6 and C3H/He strains, on the other hand, developed severe uterine edema after 4 weeks of E2 exposure, preventing the onset of EH in these strains (Fig. S1). Although the mechanisms by which E2 triggers uterine edema in C57BL/6 and C3H/He are not fully understood, it might be due to the recruitment of different immune cells 26. We extracted and analyzed the uterine edema fluids formed in C3H/He and C57BL/6 mouse uteri using flow cytometry. The result revealed two distinct types of immune cells in the edema fluids as shown in Figure S2. All these results support the conclusion that this newly introduced animal model may be mostly suitable to be conducted in Balb/c mice for long-term studies.

Identifying ER and PR status in EH tissues can provide crucial information about the susceptibility of a tissue to hormones, and thus, predict response to therapy. Since expression levels of PR and ER can be distinctly different between EH and EC, we evaluated their expression to assess the malignancy potential in mouse endometrial tissues. PR expression in glandular epithelium was lower in simple EH when compared to atypical type. Although PR and ER expressions were the strongest in atypical EH, a decrease or in some cases even a complete disappearance of PR expression was seen in some glands as atypical EH progressed to borderline EC. Loss of hormone receptors expression is known to be an early event in EC, and the absence of receptors is usually associated with less differentiated high-grade cancers. It is known that E2 increases the levels of both ER and PR within the endometrium. Since the expression of steroidal hormone receptors in our model occurred in response to E2, this may indeed reinforce the fact that EH is mainly hormonally driven 27. Expression of both ER and PR receptors in this study were consistent with results seen in hyperplastic human endometrial tissues. High ER and PR levels existed in association with atypical features, and are known to decrease as hyperplasia advances to EC 28,29.

The association between local tissue inflammation and cancer development has been discussed extensively 30,31. Although the exact mechanisms by which these inflammatory responses promote the onset and progression of cancer still remain a subject of considerable debate, inflammatory cells are known to cause an increase in cell proliferation with the presence of free radicals leading to DNA damage and/or proteins modification 32. Inflammatory responses exist within the endometrium as normal physiological components of the menstrual cycle. CD45+ cells are scattered throughout the endometrial stroma and they start to aggregate around endometrial glands during late secretory phase of the cycle following a drop in progesterone levels. In case of estrogen dominance, the production of proinflammatory components such as IL-6, TNF *α*, matrix metalloproteinases, can participate in the neoplastic transformation and carcinogenesis of the endometrium 33. Progenitor CD45+ cells increase during the abnormal proliferation of the endometrium, with mostly lymphocyte-like appearance, but it is currently not known whether their increase stimulates the formation of new epithelial cells, or is just a consequence of the presence of hyperplastic lesions and inflammatory events in the endometrium 33–35. The notion that different types of cancers like colon, prostate, and gastric are preceded with inflammatory responses might be in favor of CD45+ cell infiltration being an inflammatory component leading to the transformation of healthy tissues to cancerous rather than being a consequence 33. In light of recent clinical findings, our results seem consistent with CD45+ patterns observed within hyperplastic endometrium where aggregation and further penetration of CD45+ cells to glandular epithelium occurred in complex atypical EH as well as borderline EC.

Besides CD45+ expression and alterations of the receptor status seen in our model, a loss in PTEN expression was also found within 10 weeks of continuous E2 exposure. PTEN decreases phospholipid phosphatidylinositol-(34,5)-triphosphate (PIP3) levels, responsible for the activation of cell proliferation, consecutively leading to the downregulation of cell proliferation 36. Previous study indicated that PTEN silencing can be detected at early stage of EH and is associated with the transformation from simple EH to atypical type 37,38. Silencing of the PTEN has been identified in histologically normal-appearing endometrium exposed to estrogen, 19% in atypical EH, 21% and nonatypical EH suggesting that PTEN aberrations are an early event in the development of EC 39. A common joint loss in the expression of PTEN and paired box containing gene, PAX2, has been seen in EH and EC. PAX2 has a crucial role in endometrial self-renewal mechanism. Other reports suggested that it acts as a tumor suppressor gene. The joint loss of PTEN and PAX2 in our model, which took a clonal pattern, occurred mostly in atypical glands. Although reports indicated that inactivation of PTEN and PAX2 occur independently, a joint silencing happening in the same gland promote malignant transformation 40.

In addition to PTEN, it was estimated that 14–44% of ECs are associated with an abnormal increase in *β*-catenin activation and translocation 39. *β*-catenin is a component of the E-cadherin-catenin unit, essential for cell differentiation and homeostasis. Moreover, it is an important member of the Wnt/*β*-catenin signaling pathway, which is required for adult tissue maintenance 39. Accumulation of *β*-catenin in the cytoplasm and the nucleus of endometrial glandular epithelium is considered to be a mutation marker in exon 3 of *β*-catenin associated with the development of EC. Previous studies showed that mutated *β*-catenin might act as a mediator for the uterine response to E2 also leading to the alteration in epithelial signaling and the development of EH 41. *β*-catenin mutation was described to be more common with atypical EH and EC than the nonatypical type 42,43. The accumulation of *β*-catenin in our model found after the development of atypical EH following 8 weeks of continuous exposure to E2 alongside other mutations is consistent with previous results seen in human patients, and support the fact that our E2-induced EH mouse model is indeed a representative of human disease.

In summary, we developed a new EH mouse model based on the use of a subcutaneous E2 sustained releasing pellet. The onset and progression of EH generated within this model successfully recapitulate pathological transformation as observed in human patients. Moreover, this model produced various clinically relevant genetic aberrations, which are crucial in the improvement of novel treatments preclinical testing for the management of EH. In comparison with other currently available EH models, this model is cost-effective, reliable, and easily reproducible, which may serve as a new preclinical research tool and facilitate the investigation of tumorigenesis mechanisms.

## Supporting Information

**Figure S1.** Severe edema was developed in mouse uteri after 4 weeks of E2 exposure. (A) The uterus of Balb/c mouse showed no significant change in size. (B) C57BL/6 and (C) C3H/He mouse uteri were filled with fluids.

**Figure S2.** Different immune cells were identified in uterine edema fluids of C3H/He and C57BL/6 mice. FACS analysis of edema fluid from (A) C3H/He and (B) C57BL/6 mouse uteri using F4/80, GR1, CD11b, CD3ε, and B220 markers. Result showed that edema fluid from C3H/He uterus contained a large amount of GR1+/CD11b+ myeloid cells.

**Table S1.** Antibody information and staining condition.

**Table S2.** The success rates for the development of different stages of EH based on time of exposure to E2.

## References

[b1] Siegel R, Ma J, Zou Z, Jemal A (2014). Cancer statistics, 2014. CA Cancer J. Clin.

[b2] Lacey JV, Chia VM (2009). Endometrial hyperplasia and the risk of progression to carcinoma. Maturitas.

[b3] Schlaen I, Bocanera R, Figueroa-Casas P (1986). Endometrial cancer and its precursors: a comparison of histological and clinical features. Maturitas.

[b4] Kirschner MA, Schneider G, Ertel NH, Worton E (1982). Obesity, androgens, estrogens, and cancer risk. Cancer Res.

[b5] Trimble CL, Method M, Leitao M, Lu K, Ioffe O, Hampton M (2012). Management of endometrial precancers. Obstet. Gynecol.

[b6] Creasman WT (1997). Endometrial cancer: incidence, prognostic factors, diagnosis, and treatment. Semin. Oncol.

[b7] Holland C (2011). Unresolved issues in the management of endometrial cancer. Expert Rev. Anticancer Ther.

[b8] Couse JF, Davis VL, Hanson RB, Jefferson WN, McLachlan JA, Bullock BC (1997). Accelerated onset of uterine tumors in transgenic mice with aberrant expression of the estrogen receptor after neonatal exposure to diethylstilbestrol. Mol. Carcinog.

[b9] Erdemoglu E, Güney M, Giray SG, Take G, Mungan T (2009). Effects of metformin on mammalian target of rapamycin in a mouse model of endometrial hyperplasia. Eur. J. Obstet. Gynecol. Reprod. Biol.

[b10] Milam MR, Celestino J, Wu W, Broaddus RR, Schmeler KM, M Slomovitz B (2007). Reduced progression of endometrial hyperplasia with oral mTOR inhibition in the Pten heterozygote murine model. Am. J. Obstet. Gynecol.

[b11] Newbold RR, Bullock BC, McLachlan JA (1990). Uterine adenocarcinoma in mice following developmental treatment with estrogens: a model for hormonal carcinogenesis. Cancer Res.

[b12] Niwa K, Tanaka T, Mori H, Yokoyama Y, Furui T, Tamaya T (1991). Rapid induction of endometrial carcinoma in ICR mice treated with *N*-methyl-*N*-nitrosourea and 17 beta-estradiol. Jpn. J. Cancer Res.

[b13] Podsypanina K, Ellenson LH, Nemes A, Gu J, Tamura M, Yamada KM (1999). Mutation of Pten/Mmac1 in mice causes neoplasia in multiple organ systems. Proc. Natl Acad. Sci. USA.

[b14] Wang H, Douglas W, Lia M, Edelmann W, Ra Kucherlapati, Podsypanina K (2002). DNA mismatch repair deficiency accelerates endometrial tumorigenesis in Pten heterozygous mice. Am. J. Pathol.

[b15] Becher OJ, Holland EC (2006). Genetically engineered models have advantages over xenografts for preclinical studies. Cancer Res.

[b16] Richmond A, Su Y (2008). Mouse xenograft models vs GEM models for human cancer therapeutics. Dis. Model. Mech.

[b17] Damsch S, Eichenbaum G, Tonelli A, Lammens L, Van BK, Feyen B (2011). Gavage-related reflux in rats: identification, pathogenesis, and toxicological implications (review). Toxicol. Pathol.

[b18] DeRose YS, Gligorich KM, Wang G, Georgelas A, Bowman P, Courdy SJ (2013). Patient-derived models of human breast cancer: protocols for in vitro and in vivo applications in tumor biology and translational medicine. Curr. Protoc. Pharmacol.

[b19] DeRose YS, Wang G, Lin Y-C, Bernard PS, Buys SS, Ebbert MTW (2011). Tumor grafts derived from women with breast cancer authentically reflect tumor pathology, growth, metastasis and disease outcomes. Nat. Med.

[b20] Jeong J-W, Lee HS, Lee KY, White LD, Broaddus RR, Zhang Y-W (2009). Mig-6 modulates uterine steroid hormone responsiveness and exhibits altered expression in endometrial disease. Proc. Natl. Acad. Sci. U. S. A.

[b21] Schwartz J, Sartini D, Huber S (2004). Myocarditis susceptibility in female mice depends upon ovarian cycle phase at infection. Virology.

[b22] Aleem FA, Moukhtar MA, Hung HC, Romney SL (1976). Plasma estrogen in patients with endometrial hyperplasia and carcinoma. Cancer.

[b23] Tsuchiya Y, Nakajima M, Yokoi T (2005). Cytochrome P450-mediated metabolism of estrogens and its regulation in human. Cancer Lett.

[b24] Theodorsson A, Hilke S, Rugarn O, Linghammar D, Theodorsson E (2005). Serum concentrations of 17beta-estradiol in ovariectomized rats during two times six weeks crossover treatment by daily injections in comparison with slow-release pellets. Scand. J. Clin. Lab. Invest.

[b25] Plymate SR, Matej LA, Jones RE, Friedl KE (1988). Inhibition of sex hormone-binding globulin production in the human hepatoma (Hep G2) cell line by insulin and prolactin. J. Clin. Endocrinol. Metab.

[b26] Watanabe H, Numata K, Ito T, Takagi K, Matsukawa A (2004). Innate immune response in Th1- and Th2-dominant mouse strains. Shock.

[b27] Goncharenko VM, Beniuk VA, Kalenska OV, Demchenko OM, Spivak MY, Bubnov RV (2013). Predictive diagnosis of endometrial hyperplasia and personalized therapeutic strategy in women of fertile age. EPMA J.

[b28] Nunobiki O, Taniguchi E, Ishii A, Tang W, Utsunomiya H, Nakamura Y (2003). Significance of hormone receptor status and tumor vessels in normal, hyperplastic and neoplastic endometrium. Pathol. Int.

[b29] Punnonen R, Mattila J, Kuoppala T, Koivula T (1993). DNA ploidy, cell proliferation and steroid hormone receptors in endometrial hyperplasia and early adenocarcinoma. J. Cancer Res. Clin. Oncol.

[b30] Asakawa MG, Goldschmidt MH, Une Y, Nomura Y (2008). The immunohistochemical evaluation of estrogen receptor-alpha and progesterone receptors of normal, hyperplastic, and neoplastic endometrium in 88 pet rabbits. Vet. Pathol.

[b31] Bernstein CN, Blanchard JF, Kliewer E, Wajda A (2001). Cancer risk in patients with inflammatory bowel disease: a population-based study. Cancer.

[b32] Hussain SP, Hofseth LJ, Harris CC (2003). Radical causes of cancer. Nat. Rev. Cancer.

[b33] Wallace AE, Gibson DA, Saunders PTK, Jabbour HN (2010). Inflammatory events in endometrial adenocarcinoma. J. Endocrinol.

[b34] Wahab M, Thompson J, Al-Azzawi F (1999). The distribution of endometrial leukocytes and their proliferation markers in trimegestone-treated postmenopausal women compared to the endometrium of the natural cycle: a dose-ranging study. Hum. Reprod.

[b35] Bratincsák A, Brownstein MJ, Cassiani-Ingoni R, Pastorino S, Szalayova I, Tóth ZE (2007). CD45-positive blood cells give rise to uterine epithelial cells in mice. Stem Cells.

[b36] Latta E, Chapman WB (2002). PTEN mutations and evolving concepts in endometrial neoplasia. Curr. Opin. Obstet. Gynecol.

[b37] Jarboe EA, Mutter GL (2010). Endometrial intraepithelial neoplasia. Semin. Diagn. Pathol.

[b38] Sakuragi N, Salah-eldin A, Watari H, Itoh T, Inoue S, Moriuchi T (2002). Bax, Bcl-2, and p53 expression in endometrial cancer. Gynecol. Oncol.

[b39] Matias-Guiu X, Catasus L, Bussaglia E, Lagarda H, Garcia A, Pons C (2001). Molecular pathology of endometrial hyperplasia and carcinoma. Hum. Pathol.

[b40] Monte NM, Webster KA, Neuberg D, Dressler GR, Mutter GL (2010). Joint loss of PAX2 and PTEN expression in endometrial precancers and cancer. Cancer Res.

[b41] Villacorte M, Suzuki K, Hirasawa A, Ohkawa Y, Suyama M, Maruyama T (2013). β-Catenin signaling regulates Foxa2 expression during endometrial hyperplasia formation. Oncogene.

[b42] Ashihara K, Saito T, Mizumoto H, Nishimura M, Tanaka R, Kudo R (2002). Mutation of beta-catenin gene in endometrial cancer but not in associated hyperplasia. Med. Electron. Microsc.

[b43] Moreno-Bueno G, Hardisson D, Sarrió D, Sarrió D, Sánchez C, Cassia R (2003). Abnormalities of E- and P-cadherin and catenin (beta-, gamma-catenin, and p120ctn) expression in endo metrial cancer and endometrial atypical hyperplasia. J. Pathol.

