# Supplementary material for: An estrogen-induced endometrial hyperplasia mouse model recapitulating human disease progression and genetic aberrations
Source: Cancer Med. 2015 Mar 23;4(7):1039–50. doi: 10.1002/cam4.445 (PMC4529342; doi:10.1002/cam4.445)
Supplement: Supplementary file 1 [file cam40004-1039-sd1.ppt]

## Slide 1
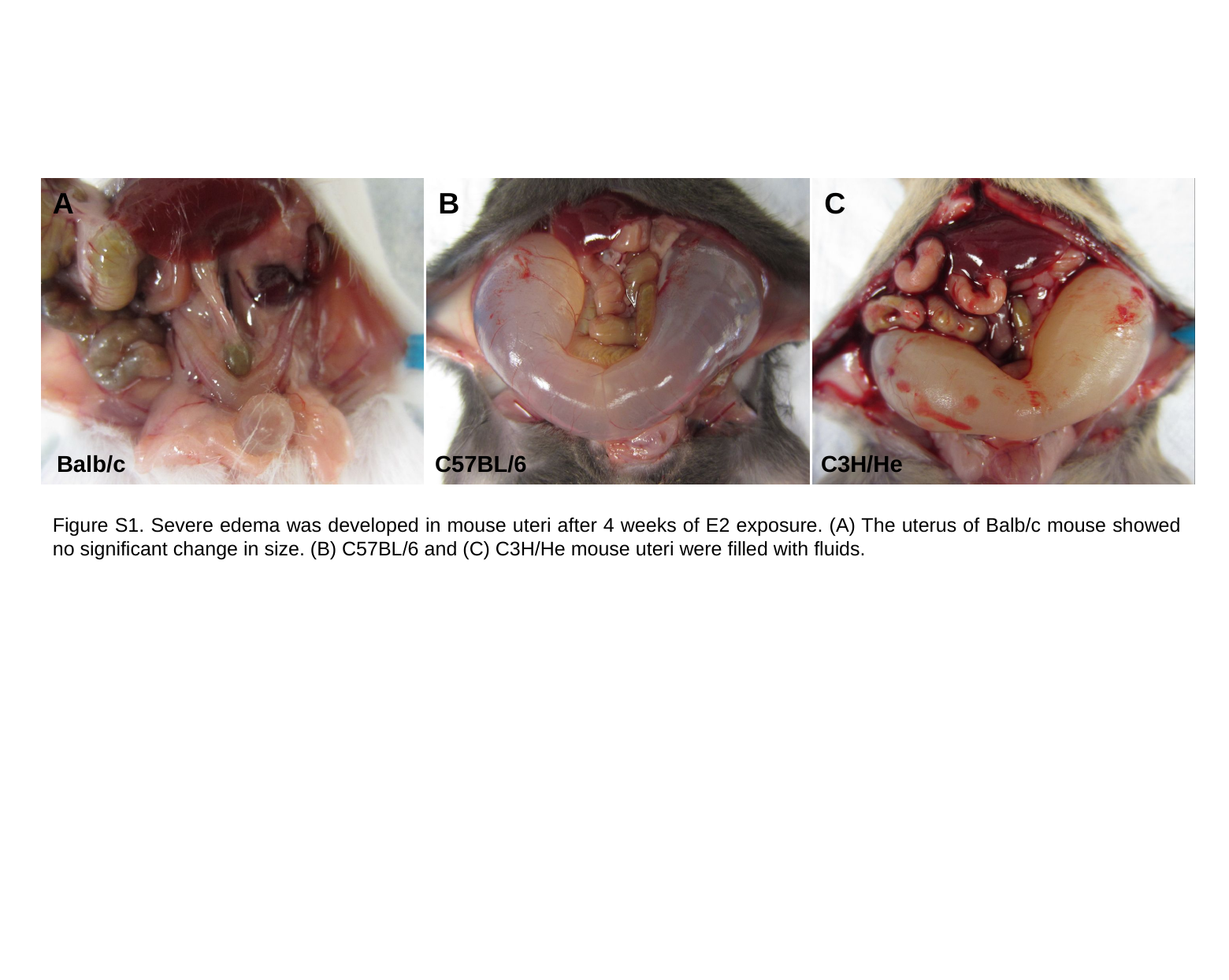

A
B
C
Balb/c
C57BL/6
C3H/He
Figure S1. Severe edema was developed in mouse uteri after 4 weeks of E2 exposure. (A) The uterus of Balb/c mouse showed no significant change in size. (B) C57BL/6 and (C) C3H/He mouse uteri were filled with fluids.
