# Supplementary material for: An estrogen-induced endometrial hyperplasia mouse model recapitulating human disease progression and genetic aberrations
Source: Cancer Med. 2015 Mar 23;4(7):1039–50. doi: 10.1002/cam4.445 (PMC4529342; doi:10.1002/cam4.445)
Supplement: Supplementary file 2 [file cam40004-1039-sd2.ppt]

## Slide 1
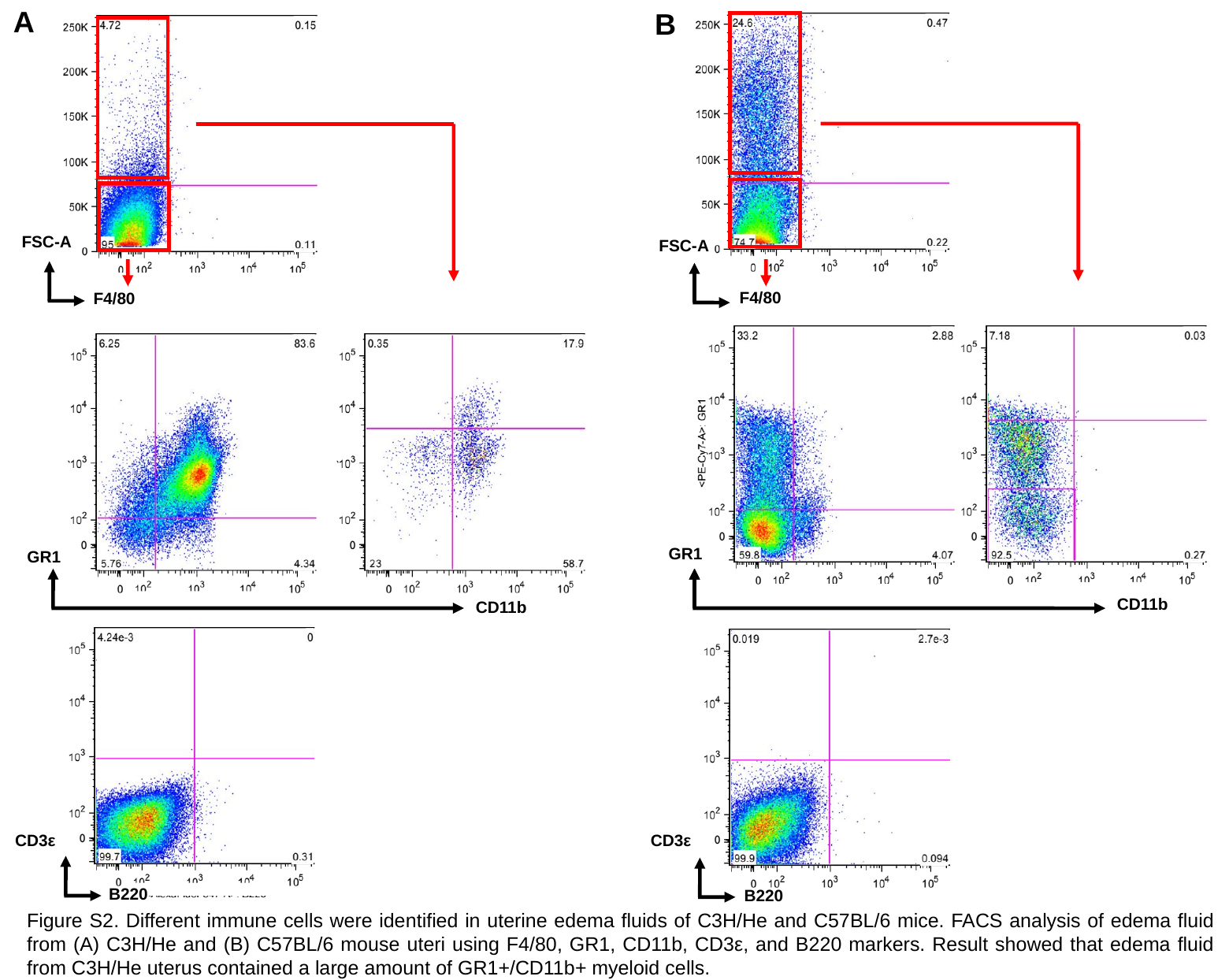

A
B
FSC-A
FSC-A
F4/80
F4/80
GR1
GR1
CD11b
CD11b
CD3ε
CD3ε
B220
B220
Figure S2. Different immune cells were identified in uterine edema fluids of C3H/He and C57BL/6 mice. FACS analysis of edema fluid from (A) C3H/He and (B) C57BL/6 mouse uteri using F4/80, GR1, CD11b, CD3ε, and B220 markers. Result showed that edema fluid from C3H/He uterus contained a large amount of GR1+/CD11b+ myeloid cells.
