# Supplementary material for: An estrogen-induced endometrial hyperplasia mouse model recapitulating human disease progression and genetic aberrations
Source: Cancer Med. 2015 Mar 23;4(7):1039–50. doi: 10.1002/cam4.445 (PMC4529342; doi:10.1002/cam4.445)
Supplement: Supplementary file 3 [file cam40004-1039-sd3.doc]

**Table S1.** Antibody information and staining condition

| **Antigen** | **Antigen Retrieval** | | **Primary Antibody Incubation** | | | **Antibody Supplier** | |
| --- | --- | --- | --- | --- | --- | --- | --- |
|  | **Buffer** | **Time** | **Host** | **Dilution** | **Incubation Time** | **Manufacturer** | **Cat no.** |
| CD45 | 10 mM sodium citrate buffer (pH 6.0) | 20 mins | Rabbit | 1: 50 | 1 hr at RT | Abcam, MA, USA | ab10558 |
| PR | 10 mM sodium citrate buffer (pH 6.0) | 30 mins | Rabbit | 1:20 | 20 mins at RT | Abcam, MA, USA | ab131486 |
| ER | 10 mM sodium citrate buffer (pH 6.0) | 20 mins | Rabbit | 1:50 | O/N at 4°C | Abcam, MA, USA | ab80922 |
| β-catenin | 10 mM sodium citrate buffer (pH 6.0) | 20 mins | Mouse | 1:100 | O/N at 4°C | Thermo Scientific, IL, USA | MA1-300 |
| PTEN | 10 mM sodium citrate buffer (pH 6.0) | 30 mins | Mouse | 1:20 | O/N at 4°C | Cascade BioScience, MA, USA | ABM-2052 |
| PAX2 | 10 mM sodium citrate buffer (pH 6.0) | 20 mins | Rabbit | 1:200 | O/N at 4°C | Abcam, MA, USA | ab38738 |
