# Supplementary material for: An estrogen-induced endometrial hyperplasia mouse model recapitulating human disease progression and genetic aberrations
Source: Cancer Med. 2015 Mar 23;4(7):1039–50. doi: 10.1002/cam4.445 (PMC4529342; doi:10.1002/cam4.445)
Supplement: Supplementary file 4 [file cam40004-1039-sd4.doc]

**Table S2.** The success rates for the development of different stages of EH based on time of exposure to E2

| **Study Point** | **Endometrial lesions No. (%)** | | |
| --- | --- | --- | --- |
|  | **Disorder Proliferation** | **Simple EH** | **Atypical EH** |
| 4 weeks | 5/5 (100%) | ---- | ---- |
| 6 weeks | 1/5 (20%) | 4/5 (80%) | ---- |
| 8 weeks | ---- | 2/5 (40%) | 3/5 (60%) |
| 10 weeks | ---- | ---- | 5/5 (100%) |
